# *Streptococcus mitis* Strains Causing Severe Clinical Disease in Cancer Patients

**DOI:** 10.3201/eid2005.130953

**Published:** 2014-05

**Authors:** Samuel A. Shelburne, Pranoti Sahasrabhojane, Miguel Saldana, Hui Yao, Xiaoping Su, Nicola Horstmann, Erika Thompson, Anthony R. Flores

**Affiliations:** MD Anderson Cancer Center, Houston, Texas, USA (S.A. Shelburne, P. Sahasrabhojane, M. Saldana, H. Yao, X. Su, N. Horstmann, E. Thompson);; Baylor College of Medicine, Houston, Texas, USA (A.R. Flores)

**Keywords:** viridans group streptococci, bacteremia, neutropenia, Pitt bacteremia score, *Streptococcus mitis*, cancer patients, cancer, multilocus sequence analysis, bacteria, characterization

## Abstract

The genetically diverse viridans group streptococci (VGS) are increasingly recognized as the cause of a variety of human diseases. We used a recently developed multilocus sequence analysis scheme to define the species of 118 unique VGS strains causing bacteremia in patients with cancer; *Streptococcus mitis* (68 patients) and *S. oralis* (22 patients) were the most frequently identified strains. Compared with patients infected with non–*S. mitis* strains, patients infected with *S. mitis* strains were more likely to have moderate or severe clinical disease (e.g., VGS shock syndrome). Combined with the sequence data, whole-genome analyses showed that *S. mitis* strains may more precisely be considered as >2 species. Furthermore, we found that multiple *S. mitis* strains induced disease in neutropenic mice in a dose-dependent fashion. Our data define the prominent clinical effect of the group of organisms currently classified as *S. mitis* and lay the groundwork for increased understanding of this understudied pathogen.

Viridans group streptococci (VGS), a genetically heterogeneous group of bacteria, are the predominant bacteria in the human oropharynx (*1*). VGS cause a wide range of infections in humans, including bacteremia in patients with neutropenia, infective endocarditis, and orbital cellulitis (*2*–*5*). However, despite the substantial clinical effect of VGS, the epidemiology and pathogenesis of these bacteria are minimally understood (*6*).

A major impediment to the study of VGS has been the inability to consistently and accurately assign VGS strains to specific species, which has resulted in numerous changes in species designation and classification schemes over time (*7*). From a clinical microbiology laboratory standpoint, automated systems have considerable limitations in VGS species identification (*8*,*9*). The problematic nature of VGS species assignment also extends to16S rRNA sequencing, the most widely used genetic tool for species identification in clinical and research settings (*9*,*10*).

Outcomes for patients with VGS bacteremia are highly variable: some patients have minimal symptoms, and others have a severe infection characterized by hypotension and acute respiratory distress syndrome (*11*). The severe infections have been termed VGS shock syndrome (*12*). Numerous studies have examined the species distribution of VGS that cause bacteremia (*9*,*13*–*16*). However, these studies have found inconsistent results between a particular VGS species and disease occurrence or clinical severity of infection (*9*,*13*,*14*,*16*,*17*). Moreover, the recently recognized limitations of previously used techniques of VGS species identification and the low number of clinical cases analyzed preclude definitive conclusions regarding the relationship between VGS species type and clinical disease (*8*,*9*,*18*). Thus, we sought to combine the species identification of a large number of VGS bloodstream isolates, which we typed by using a recently developed multilocus sequence analysis (MLSA) technique (*19*), with patient-specific clinical data to determine relationships between VGS species and clinical endpoints.

## Materials and Methods

### Study Cohort and Data Abstraction

The study cohort comprised patients at MD Anderson Cancer (MDACC) who had VGS isolated from their blood between July 1, 2011, and December 1, 2012. MDAAC is a 600-bed referral cancer hospital in Houston, Texas, USA. We used a standardized data collection form to abstract clinical data from the comprehensive electronic medical records of patients with blood culture results positive for VGS. Antimicrobial drug resistance was determined in accordance with guidelines of the Clinical and Laboratory Standards Institute (www.clsi.org/standards/). VGS are known to contaminate blood cultures and to cause clinically minor, transient bacteremia, and differentiating between contamination and infection is problematic (*20*). Thus, for the purpose of this study, we considered that patients without signs or symptoms of infection had clinically minor bacteremia, even though they may represent cases of blood culture contamination.

Severity of infection, as measured by the Pitt bacteremia score, was determined as described (*21*). Pitt bacteremia scores were not determined for patients with polymicrobial bacteremia. VGS shock syndrome was defined by using the accepted definition for septic shock (i.e., hypotension refractory to fluid replacement in the setting of an infection) (*22*). A focus of the bloodstream infection was defined as isolation of a VGS species from a nonsterile site (e.g., liver abscess) at the same time that VGS were isolated from the blood, with the exception of infective endocarditis, which was defined according the modified Duke criteria (*23*,*24*). Neutropenia was defined as an absolute neutrophil count of <500 cells/μL.

Some patients had signs and symptoms of a lower respiratory infection and x-ray findings compatible with a pneumonic process that could not be explained (i.e., no known respiratory pathogens were isolated and no other alternative explanation, e.g., congestive heart failure, was found). Such patients were defined as having unexplained pulmonary infiltrates. Because VGS are considered normal flora, isolation of these organisms from a respiratory specimen would not have been considered clinically meaningful by the clinical microbiology laboratory and thus would not have been reported. The study protocol was approved by the MDCC institutional review board.

### VGS Species Type Determination and Whole-Genome Sequencing

Bacterial isolates were identified as VGS on the basis of the following: presence of α-hemolysis, gram-positive reaction, coccus morphology arranged in chains, negative catalase test results, and exclusions of pneumococcus and enterococci by routine biochemical tests (i.e., optochin, bile solubility, and pyrrolidonyl arylamidase tests) (*25*). VGS species was determined as described (*19*). In brief, concatenated sequences of 7 housekeeping genes were used for phylogenetic tree construction in MEGA5 (megasoftware.net/); strains were assigned to VGS species on the basis of their distance from species type strains (*19*). For whole-genome sequencing of 9 *Streptococcus* *mitis* strains and 1 *S. oralis* strain, we fragmented 3 μg of genomic DNA to 350 bp (mean fragment size) and prepared barcoded sequencing libraries. The 03/10libraries were sequenced on the HiSeq 2000 sequencing System (Illumina, San Diego, CA, USA) by using 76-bp, paired-end sequencing. The raw reads in FASTQ format were aligned to the *S. mitis* B6 (GenBank accession no. NC_013853.1) and *S. pneumoniae* TIGR4 (GenBank accession no. NC_003028.3) genomes by using Mosaik (*26*). There was an average of 250× coverage per base, indicating extremely high confidence for base calls. Contigs were generated by feeding the raw genome sequence data into the A5 pipeline (*27*). Gene annotations were obtained by uploading contigs to the Rapid Annotation using the Subsystem Technology server at the National Microbial Pathogen Data Resource website (*28*). (Individual gene sequencing data have been deposited into GenBank. Short-read sequencing data have been deposited to the Short Read Archive (www.ncbi.nlm.nih.gov/Traces/sra/sra.cgi?view=announcement) under accession no. PRJNA240080.)

### Mouse Infection Studies

Experiments in mice were performed according to a protocol approved by the MDACC Institutional Animal Care and Use Committee. To induce neutropenia, we injected 5-week-old female Balb/C mice intraperitoneally with 100 mg/kg of cyclophosphamide (Sigma, St. Louis, MO, USA) on days −4 and −1 before bacterial injection. On day 0, mice (10 per bacterial dose) were injected with 100 μL of phosphate-buffered saline (PBS) containing 10-fold increments of bacteria ranging from 10^3^ to 10^7^ CFUs. As a control, 10 mice were injected with PBS alone. Mice were monitored over 7 days for near-death status. The dose at which 50% of the mice nearly died (hereafter referred to as LD_50_) was calculated by using the probit method. Neutropenia was confirmed in select mice on postinfection days 1, 3, and 6.

### Statistical Analysis

Differences between categorical variables were assessed by using the χ^2^ test; Fisher exact test was used when at least 1 category had <5 occurrences. The relationship between VGS species and Pitt bacteremia scores was analyzed by using the Mann-Whitney U test. The Bonferroni method was employed to account for multiple comparisons when appropriate. All tests of significance were 2-sided, and statistical significance was defined at p<0.05. SPSS Statistics version 19 (IBM, Armonk, NY, USA ) was used for statistical analysis.

## Results

### Study Cohort

A total of 118 consecutive patients with VGS-positive blood cultures were included in the study cohort; ≈80% of the patients had neutropenia and hematologic malignancies (Table 1). Most patients had bacteremia without a defined focus, but several other clinical scenarios were observed, including skin/soft tissue infections, gastrointestinal infections, and infective endocarditis. Most patients had clinically mild infections (Pitt bacteremia score of 0 or 1), but 25% of patients had moderate to severe infections (Pitt bacteremia scores of >2), including 12 patients who had VGS shock syndrome.

**Table 1:** Characteristics of 118 participants in a study of *Streptococcus mitis* strains causing severe clinical disease in patients with cancer*

Characteristic	No. (%)
Sex	
M	64 (54)
F	54 (46)
Mean age, y (SD, range)	50 (18, 10–79)
Malignancy	70 (59)
Leukemia/myelodysplastic syndrome	20 (17)
Hematopoietic stem cell transplantation	10 (8)
Lymphoma/myeloma	18 (15)
Solid tumor	20 (17)
Neutrophils <500/μL	95 (81)
Clinical syndrome	
Primary bacteremia	95 (80)
Gastrointestinal focus	8 (7)
Skin/soft tissue focus	4 (3)
Infective endocarditis	2 (2)
Clinically minor bacteremia	9 (7)
Polymicrobial infection	22 (19)
Pitt bacteremia score†‡	
0	35 (37)
1	36 (38)
2	5 (5)
3	7 (7)
>4	13 (13)
Antimicrobial drug susceptibility	
Penicillin	54 (46)
Ceftriaxone	107 (91)
Moxifloxacin	60 (51)
Tetracycline	69 (59)

*All patients had viridans group streptococci bacteremia. Unless otherwise noted, data are no. (%) of patients. †Severity of infection was measured by the Pitt bacteremia score as described (*21*). Scores of 0 or 1 indicate clinically mild infections; scores of >2 indicate moderate to severe infections. ‡Determined only for patients with monomicrobial infection.

### VGS Species and Bacteremia

**Figure 1:**
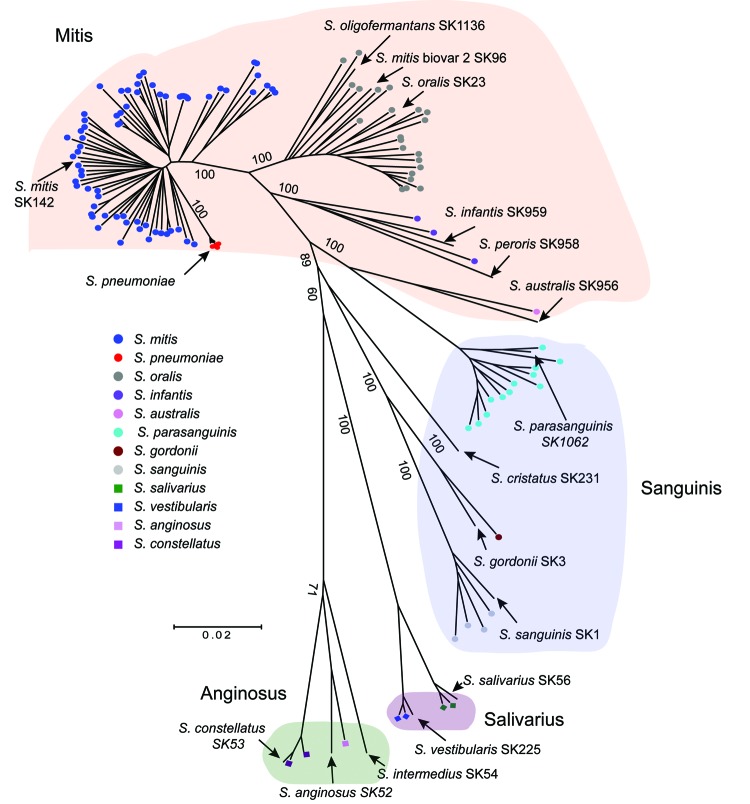
Multilocus sequence analysis (MLSA) of viridans group streptococci (VGS) strains causing bacteremia in patients with cancer. The neighbor-joining radial tree was generated by using concatenated sequences. Strains were assigned to a particular VGS on the basis of their proximity to type strains. Locations of well-characterized or type VGS strains (lines without circles) are also shown for reference purposes. Five contemporaneous *Streptococcus pneumoniae* strains are also included for reference purposes (shown in red). Numbers indicate bootstrap support values (%).Scale bar indicates genetic distance. Background colors indicate VGS species group, using the system from Sinner et al. (*29*).

To gain insight into the species of VGS causing bacteremia in the study cohort, we performed MLSA of 7 housekeeping genes, as described (*19*). Strains were assigned to species by comparing their position on the phylogenetic tree with those of established type strains (*19*). The 118 strains could be confidently assigned to 11 distinct species (Figure 1; Technical Appendix 1). The most commonly observed species were *S. mitis* (68 strains), *S. oralis* (22 strains), and *S. parasanguinis* (12 strains). For classification purposes, various VGS species are often placed into distinct groups; the association between VGS strains causing bacteremia and group assignment is shown in Figure 1, using the scheme from Sinner et al. (*29*). In total, 80% of strains were from the Mitis group, and the remaining strains were from the Sanguinis group (14%), Anginosus group (3%), and Salivarius group (3%).

### VGS Species and Clinical Syndromes

Because of the diverse genetic nature of the various VGS species, we next tested the hypothesis that distinct VGS species cause different clinical syndromes. Given the number of strains for each species, we analyzed the Mitis group species (i.e., *S. mitis* and *S. oralis*) individually and analyzed species comprising the Sanguinis, Anginosus, and Salivarius groups by group (Table 2). Compared with strains of other VGS species, *S. mitis* strains were significantly more likely to cause primary bacteremia (p<0.01) and less likely to cause polymicrobial bacteremia (p = 0.01) and clinically minor bacteremia (p<0.01). *S. oralis* strains were more likely to cause polymicrobial infection (p = 0.02), Sanguinis group strains were more likely to cause clinically minor bacteremia (p<0.01), and Anginosus group strains were significantly associated with bacteremia with a gastrointestinal focus (p<0.01). When we only considered patients with neutropenia or cases of monomicrobial bacteremia, we observed the same statistically significant species–clinical disease relationships (data not shown).

**Table 2:** Association between clinical syndrome and infecting species for 118 patients with viridans group streptococci bacteremia

Clinical syndrome	Viridans group streptococci*					
	*mitis*	*oralis*	*infantis/australis*	Sanguinis	Anginosus	Salivarius/Vestibularis
Primary bacteremia with neutropenia	58	6	3	3	0	2
Primary bacteremia without neutropenia	1	2	0	3	0	0
Gastrointestinal focus	1	2	0	1	3	1
Skin/soft tissue focus	2	1	0	1	0	0
Infective endocarditis	0	2	0	0	0	0
Polymicrobial bacteremia	6	8	0	4	0	0
Clinically minor bacteremia	0	1	1	5	0	2

*mitis, o*ralis*,*infantis*, and a*ustralis* refer to viridans group streptococci species; Sanguinis, Anginosus, Salivarius, and Vestibularis refer to viridans streptococci groups (Figure 1) (*29*).

### VGS Species and Disease Severity

**Figure 2:**
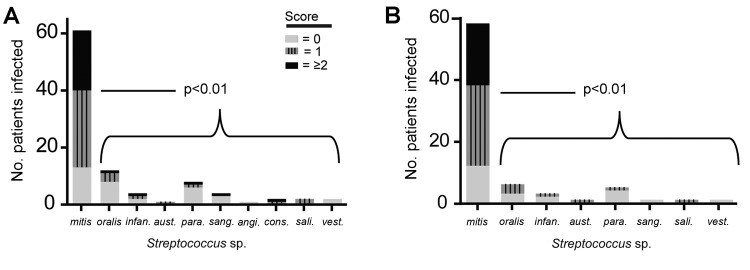
A) Pitt bacteremia scores for cancer patients infected with particular VGS species, showing that more clinically severe disease is caused by *Streptococcus mitis* strains than other viridans group streptococci (VGS) species. B) Pitt bacteremia scores for only those cancer patients with neutropenia. p values refer to Mann-Whitney U comparison of Pitt bacteremia scores for patients infected with *S. mitis* strains versus those infected with non–*S. mitis* strains. *infan.*, *infantis*; *aust*., *australis*; *para*., p*arasanguinis;* *sang*., *sanguinus*; *angi*., *anginosus*; *cons*., *constellatus*; *sali*., *salivarius*; *vest*., *vestibularis*.

We next sought to determine if there was a relationship between VGS species and disease severity (as determined by Pitt bacteremia score). Organ dysfunction, such as hypotension, begins to occur at Pitt bacteremia scores of >2 (*21*). The distribution of Pitt bacteremia score by infecting species is shown in Figure 2, panel A. Patients infected with *S. mitis* were significantly more likely to have a higher Pitt bacteremia score (p<0.01). One possible explanation for this observation is that *S. mitis* strains mainly caused infections in patients with neutropenia who, compared with patients without neutropenia, might be more likely to have serious infections. Thus, we repeated the analysis, including only patients with neutropenia. Again, the Pitt bacteremia scores were significantly higher for patients infected with *S. mitis* (p<0.01; Figure 2, panel B). Of the 12 cases of VGS shock syndrome, 11 were caused by *S. mitis* strains and 1 was caused by an *S. constellatus* strain (Anginosus group).

### Identification of *S. mitis* Strain Clusters

**Figure 3:**
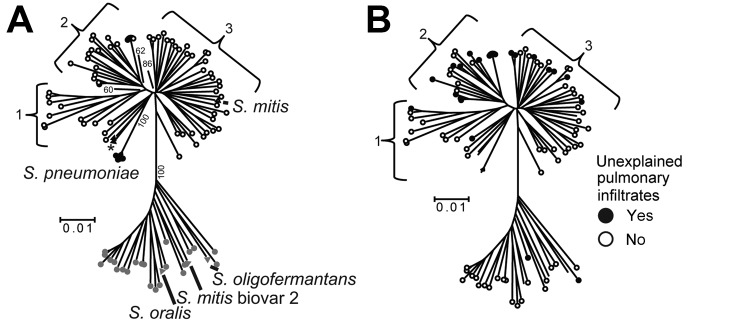
Multilocus sequence analysis (MLSA) and clinical correlates of *Streptococcus mitis* and *S. oralis* strains. A) For reference purposes, the following are labeled: viridans group streptococci (VGS) strains (SK142 for *S. mitis*, SK23 for *S. oralis*, SK1136 for *S. oligofermantans*), 5 *S. pneumoniae* strains, 2 *S. pseudopneumoniae* strains (SK674 and 103, indicated by an asterisk), and strain SK96 (previously characterized as an *S. mitis* biovar 2 strain). Numbers within the tree refer to bootstrap support values (%).B) For reference purposes, branches of the previously labeled VGS, *S. pneumoniae*, and *S. pseudopneumoniae* strains have been retained; however, for clarity, the branches are not labeled. The presence or absence of unexplained pulmonary infiltrates is indicated as described in the key. Bootstrap support values are the same as in panel A. Numbers 1–3 indicate *S. mitis* clusters, and scale bars indicate genetic distances.

Most cases of bacteremia and severe disease occurred in patients infected with *S. mitis*; thus, we focused on *S. mitis* strains and strains from the closely related *S. oralis* species. In contrast to what we observed for the *S. oralis* strains, several distinct groupings could be visualized within the *S. mitis* strains, which we arbitrarily labeled as clusters 1, 2, and 3 (Figure 3, panel A). *S. mitis* cluster 1 comprised 10 strains, including 2 that were genetically identical by MLSA, and cluster 2 comprised 22 strains, including 6 that were genetically identical. Cluster 3 comprised 29 strains and may contain additional strain groupings, but further phylogenetic delineation of this cluster could not be done with sufficient confidence. We did not observe substantial differences, in terms of distinct disease types or severity of infection, between patients from whom the *S. mitis* cluster strains were derived (Technical Appendix 2). However, there was a predominance of unexplained pulmonary infiltrate cases among patients infected with cluster 2 strains (p<0.01 for cluster 2 strains vs. noncluster 2 strains) (Figure 3, panel B). This variable was investigated because, given the close genetic relationship between *S. mitis* and *S. pneumoniae*, we hypothesize that *S. mitis* strains may cause pneumonia in severely immunocompromised persons (Figure 1).

### Whole-Genome Analysis and MLSA Grouping of Strains

**Figure 4:**
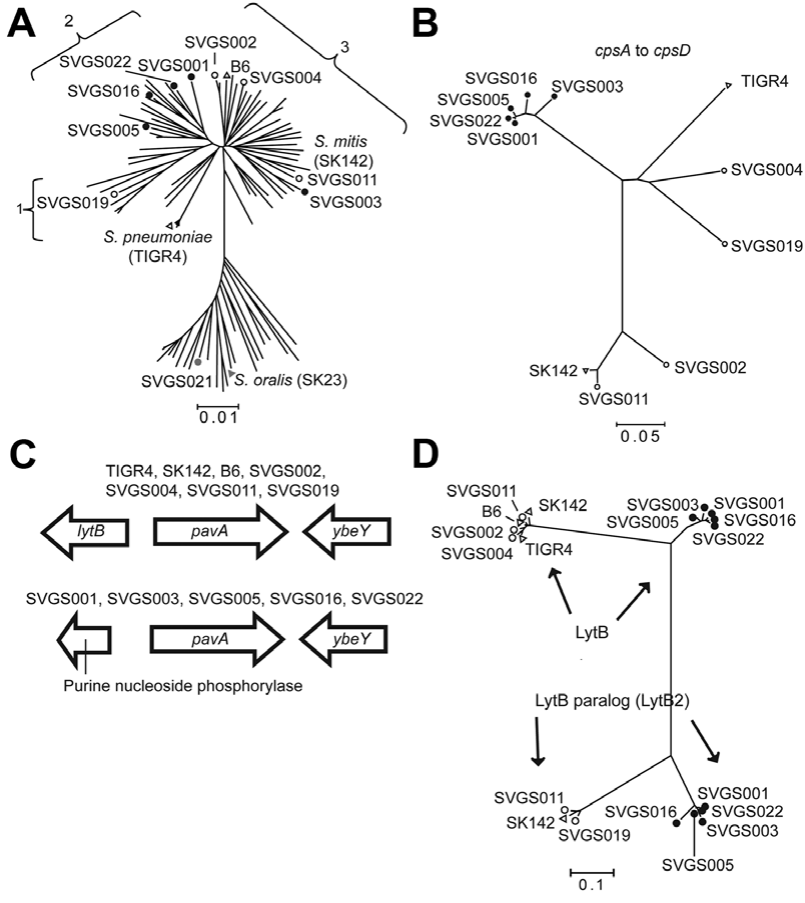
Selected data from whole-genome analysis of viridans group streptococci (VGS) strains. A) Neighbor-joining tree generated by multilocus sequence analysis (MLSA) of *Streptococcus mitis* and *S. oralis* strains, showing locations of VGS strains selected for whole-genome analysis. Numbers 1–3 refer to *S. mitis* clusters (defined in Figure 3). MLSA locations are also shown for the *S. mitis* and *S. oralis* type strains (SK142 and SK23, respectively) and fully sequenced *S. mitis* strain B6 and *S. pneumoniae* strain TIGR4. B) SVGS004 mouse challenge data. Neighbor-joining tree of first 4 genes of the capsular polysaccharide encoding operon (*cpsA*–*cpsD*). TIGR4 and SK142 are included for reference purposes. Strain B6 is not included because it lacks a *cps* operon. Note tight clustering of 5 VGS strains (black dots). C) Genetic arrangement surrounding the *pavA* gene, which encodes a fibronectin-binding protein. Two distinct gene arrangements are present 5′ of the *pavA* gene, with the arrangement for particular strains as indicated. D) Neighbor-joining tree of LytB protein, which is involved in cell-wall turnover, from fully sequenced *S. mitis* strains. Some *S. mitis* strains possess a gene encoding a second LytB-like protein, which we have named LytB2 (ZP_07643922 from strain SK142). Note tight clustering of the same 5 VGS strains (black dots) for the LytB and LytB2 proteins as was observed for the *cpsA*–*cpsD* analysis in panel B. A, B, D) SVGS, Shelburne VGS. Scale bars indicate genetic distances.

To determine whether the MLSA data accurately represented the entire genetic content of the Mitis group strains, we performed whole-genome sequencing of 9 *S. mitis* and 1 *S. oralis* isolates (Figure 4, panel A). For the 9 *S. mitis* strains, the reads mapped to ≈70% coverage of the only completely finished *S. mitis* genome (*S. mitis* strain B6 [*30*]), Technical Appendix 2, Table 1). This considerable level of intraspecies genetic diversity for *S. mitis* strains has been observed previously in sequencing and DNA:DNA hybridization studies and meant that we could not use whole-genome analysis of single-nucleotide polymorphisms to determine strain relatedness (*30*,*31*). Thus, we next sought to identify regions of genetic similarity among the strains that could be analyzed for interstrain comparisons.

All 9 *S. mitis* strains contained operons encoding a putative polysaccharide capsule. The first 4 genes of the operon, corresponding to *cpsA*–*cpsD* in *S. pneumoniae,* were relatively well conserved among the 9 strains. Concatenated alignment of *cpsA*–*cpsD* showed a close relationship for the 4 cluster 2 strains and strain Shelburne VGS (SVGS) 003, whereas the *cpsA*–*cpsD* genes from the remaining 4 strains were more closely related to the *S. pneumoniae* strain TIGR4 (SVGS004 and SVGS019) or to the *S. mitis* type strain SK142 (SVGS002 and SVGS011) (Figure 4, panel B).

In addition to the capsule operons, multiple other comparisons arising from our whole-genome analysis confirmed the idea that the 4 cluster 2 strains and strain SVGS003 were closely related. All of the *S. mitis* strains contained the gene encoding the fibrinogen-binding protein, PavA (pneumococcal adherence and virulence protein A). However, there was a different gene 5′ to the *pavA *gene in SVGS003 and the 4 cluster 2 strains than in the other 4 *S. mitis* strains (Figure 4, panel C). In a similar manner, the LytB protein in cluster 2 strains and SVGS003 grouped separately from the LytB protein in the other strains, and a LytB paralog in strain SVGS003 and the 4 cluster 2 strains was distinct from other forms of the LytB protein and from LytB paralogs encoded by SVGS011 and SVGS019. 

LytB is part of a group of choline-binding proteins that are involved in cell-wall turnover, some of which have been shown to be important for virulence in *S. pneumoniae* (*32*). When the presence or absence of choline-binding proteins was determined for the various strains, substantial strain-to-strain heterogeneity was observed (Technical Appendix 2 Table 2). The only repeating pattern of gene content was 1 that occurred for 3 different choline-binding protein–encoding genes: *cbpE, cbpI,* and *lytC*. These 3 genes, which are present in diverse chromosomal locations, were absent in the 4 cluster 2 strains, SVGS003, and the *S. oralis* strain SVGS021 but present in the other noncluster 2 *S. mitis* strains. Thus, the whole-genome data support MLSA data, assigning 4 of the fully sequenced strain to *S. mitis* cluster 2; strain SVGS003, a group 3 strain by MLSA, appears to have genetic characteristics of the cluster 2 strains by whole-genome analysis.

### Mouse Model for Testing VGS Virulence

**Figure 5:**
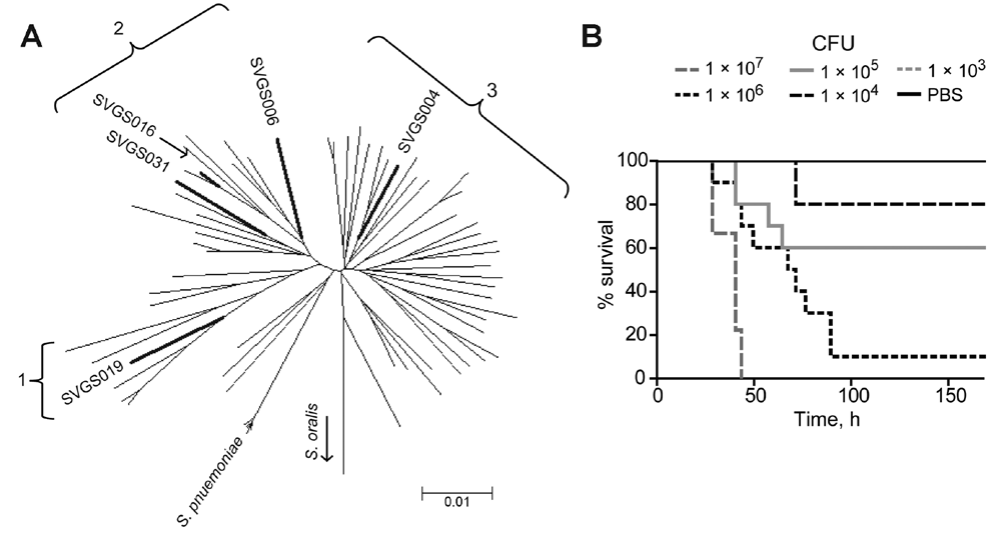
*Streptococcus mitis* strains cause dose-dependent disease in mice with neutropenia. A) Multilocus sequence analysis–generated neighbor-joining tree showing genetic relationships among all *S. mitis* strains. Bold branches indicate locations of the 5 strains used in the mouse model challenge experiment. Numbers 1–3 refer to clusters of *S. mitis* strains (defined in Figure 3). Scale bar indicates genetic distance. B) Example of mouse challenge data. Ten neutropenic Balb/C mice per dose were infected intraperitoneally with serial 10-fold CFU dilutions of strain SVGS004 (range 10^7^–10^3^) and monitored for 168 h (7 d) for near-death status. Phosphate buffered saline (PBS) was injected as a control. None of the mice injected with PBS or with the 10^3^ dose were near death; thus, the 1 × 10^3^ line is obscured by the PBS line on the graph. SVGS, Shelburne viridans group streptococcus.

Given the apparent differences in genetic content among *S. mitis* strains, we sought to develop an animal model for testing VGS virulence that would approximate the disease observed in cancer patients with neutropenia. No neutropenia model of VGS infection exists, so we used serial 10-fold CFU dilutions of 5 *S. mitis* strains to determine the LD_50_ of organisms for the endpoint of being near death. The *S. mitis* challenge strains included isolates from the major *S. mitis* clusters (Figure 5; Table 3), and we also injected PBS as a control. None of the mice injected with PBS became ill, indicating that neither the neutropenia nor the injection itself caused major disease (Figure 5, panel B). All of the *S. mitis* strains could cause near-death status, and a dose–response relationship was observed for all strains (see example in Figure 5, panel B), but the LD_50_ varied by 100-fold among the strains (Table 3). Strain SVGS016, which caused the most severe clinical disease (i.e., it was isolated from a patient with the highest Pitt bacteremia score) also was the most virulent in the mouse model. Thus, we suggest that *S. mitis* strains cause disease in mice with neutropenia and that there is differential virulence in this mouse model among genetically diverse *S. mitis* strains.

**Table 3:** Relative virulence of viridans group streptococci strains in a neutropenic mouse model*

Strain	*Streptococcus mitis* cluster	Pitt bacteremia score of infected patient	LD_50_
SVGS004	3	1	4.1 × 10^5^
SVGS006	2	2	1.6 × 10^6^
SVGS016	2	1	1.4 × 10^5^
SVGS019	1	3	1.9 × 10^4^
SVGS031	2	1	3.6 × 10^5^

*SVGS, Shelburne viridans group streptococcus; LD_50_, considered, for the purpose of this study, to be the dose at which 50% of the animals nearly died.

## Conclusions

Since first being identified as causative agents of infections in cancer patients with neutropenia ≈35 years ago (*33*), VGS have come to be appreciated as major bacterial pathogens in patients with malignancy (*2*,*12*,*14*,*34*,*35*). The emergence of VGS as common infectious agents has coincided with the increasing use of prophylactic antimicrobial drugs, especially fluoroquinolones, for patients with neutropenia (*36*). However, despite the clear clinical consequences of VGS infections, there is minimal understanding of their pathophysiology.

A critical first step in the study of VGS is to define the clinical syndromes caused by various VGS species. This goal has long been hampered by difficulties in using phenotypic methods or single-gene sequencing approaches to assign VGS strains to particular species (*18*). Through the use of a recently developed MLSA approach (*19*), we showed that there is a relationship between VGS species, as defined genetically, and disease manifestations in patients with cancer (Table 2). An unexpected finding was the relationship between Sanguinis group strains and clinically minor bacteremia; Sanguinis group species are the leading VGS cause of infective endocarditis and have been reported to cause virulent infections in patients with neutropenia (*17*,*37*). One possible explanation for this finding is that Sanguinis group VGS are often causative agents of transient bacteremia and that transient bacteremia occasionally results in infective endocarditis. Platelets are thought to be critical to the pathogenesis of VGS infective endocarditis. Thus, because of low platelet counts, persons with cancer, especially those with hematologic malignancy, may be relatively resistant to the development of infective endocarditis after transient VGS bacteremia (*38*).

Another key relationship that we observed was that of *S. mitis* and primary bacteremia during periods of neutropenia. Our data support and extend the findings of other smaller studies using genetic techniques that found a similar predominance of *S. mitis* strains in patients with neutropenia (*9*,*15*,*39*). The reason that *S. mitis* strains are the leading cause of VGS bacteremia in patients with neutropenia is not known. One could postulate that *S. mitis* is simply the dominant commensal VGS species and thus is the most likely species to translocate across epithelial barriers when patients become neutropenic. Indeed, a recent microbiome study showed that *S. mitis* is the predominant VGS species isolated from buccal mucosa samples from healthy persons (*1*). However, in our study, *S. mitis* not only caused the majority of neutropenic infections but also caused a disproportionate percentage of serious infections (Figure 2). Thus, at least for patients with neutropenia, *S. mitis* is more likely than other VGS to enter into the bloodstream and to cause serious infections once there. Moreover, compared with other VGS species, *S. mitis* rarely caused clinically minor bacteremia or polymicrobial infection, suggesting that *S. mitis* strains have inherently virulent properties compared with other VGS. The data from our multistrain, whole-genome sequencing and the development of an animal model of neutropenia and *S. mitis* infection should provide a key platform for elucidating *S. mitis* virulence.

The deep branching pattern produced by MLSA of our *S. mitis* strains isolated from human blood has been observed in other investigations (*19*,*30*) and suggests that the organisms currently grouped as *S. mitis* may more precisely be considered as >2 species. The application of whole-genome sequencing to large numbers of *S. mitis* strains will be necessary to fully resolve *S. mitis* strain clusters, as shown by the somewhat discordant results of our MLSA and whole-genome analysis. In addition, we were intrigued by the association of cluster 2 *S. mitis* strains and unexplained pneumonia (Figure 3, panel B). Given the close genetic relationship between *S. mitis* and *S. pneumoniae*, it might be expected that some *S. mitis* strains could cause pneumonia, especially in severely immunocompromised patients. Whether particular subspecies of *S. mitis* can cause pneumonia is an active area of investigation in our laboratory, and if it does, that could help explain the stubbornly low number of pathogens that can be identified for patients with pneumonic syndromes (*40*).

This large series of invasive VGS strains includes detailed molecular and clinical information. By combining these 2 sets of data, we have definitively established the critical role of *S. mitis* strains in invasive VGS infection in patients with cancer and have laid the groundwork for future insights into how these organisms cause serious disease in vulnerable hosts.

Technical Appendix 1 Species assignment, clinical data, and individual gene data for viridans group streptococci bloodstream isolates from patients with cancer.

Technical Appendix 2 Alignment of 76-bp, paired-end reads for viridans group streptococci (VGS) strains with *Streptococcus mitis* and *S. pneumoniae* reference genomes, choline-binding proteins among sequenced VGS strains and *S. pneumoniae* strain TIGR4, and multilocus sequence analysis and clinical correlates of *S.* *mitis* and *S. oralis* strains.
