# Supplementary material for: Streptococcus mitis Strains Causing Severe Clinical Disease in Cancer Patients
Source: Emerg Infect Dis. 2014 May;20(5):762–71. doi: 10.3201/eid2005.130953 (PMC4012796; doi:10.3201/eid2005.130953)
Supplement: Technical Appendix 1 — Species assignment, clinical data, and individual gene data for viridans group streptococci bloodstream isolates from patients with cancer. [file 13-0953-Techapp-s1.pdf]

Article DOI: http://dx.doi.org/10.3201/eid2005.130953

Streptococcus mitis Strains Causing Severe Clinical Disease in Cancer Patients

Technical Appendix 2

Technical Appendix 2 Table 1. Summary of alignment of 76-bp, paired-end reads for viridans group streptococci strains with *Streptococcus mitis* and *S. pneumoniae* reference genomes*

Strain	*S. mitis* cluster by MLSA	% Coverage of *S. mitis* B6 genome	% Coverage of *S. pneumoniae* TIGR4 genome
SVGS001	2	66.20	59.00
SVGS002	3	69.90	64.10
SVGS003	2	67.60	59.60
SVGS004	3	68.60	62.50
SVGS005	2	67.00	59.10
SVGS011	3	70.20	62.20
SVGS016	2	68.30	60.30
SVGS019	1	70.10	62.50
SVGS021	NA	65.80	57.70
SVGS022	2	68.30	59.90

*SVGS, Shelburne viridans group streptococcus; NA, not applicable because SVGS021 is an *S. oralis* strain.

Technical Appendix 2 Table 2. Presence of genes encoding selected choline-binding proteins among sequenced viridans group streptococci strains and the *Streptococcus pneumoniae* strain TIGR4*

	Gene										
Strain†	*cbpI*	*cbpC*	*cbpJ*	*cbpF*	*cbpL*	*cbpE*	*lytB*	*lytC*	*lytA*	*lytB2*	*cbpD*
TIGR4	SP_0069	SP_0377	SP_0378	SP_0391	SP_0667	SP_0930	SP_0965	SP_1573	SP_1937	NP	SP_2201
SVGS001	No	Yes	Yes	Yes	Yes	No	Yes	No	No	Yes	Yes
SVGS002	Yes	No	Yes	Yes	Yes	Yes	Yes	Yes	Yes	No	Yes
SVGS003	No	No	No	Yes	Yes	No	Yes	No	No	Yes	Yes
SVGS004	Yes	No	No	Yes	Yes	Yes	Yes	Yes	No	No	Yes
SVGS005	No	No	No	Yes	Yes	No	Yes	No	No	Yes	Yes
SVGS011	Yes	No	No	No	Yes	Yes	Yes	Yes	Yes	Yes	Yes
SVGS016	No	Yes	Yes	No	Yes	No	Yes	No	No	Yes	Yes
SVGS019	Yes	Yes	Yes	Yes	Yes	Yes	No	Yes	No	Yes	Yes
SVGS021	No	Yes	Yes	No	No	No	No	No	No	Yes	Yes
SVSG022	No	Yes	Yes	Yes	Yes	No	Yes	No	No	Yes	Yes

*SVGS, Shelburne viridans group streptococcus; NP, not present in strain TIGR4. *cbp*I, *cbpE*, and l*ytC* are shaded to show similar pattern of gene presence/absence among the sequenced strains.
†All SVSG022 strains are *S. mitis* strains, except SVGS021, which is an *S. oralis* strain.

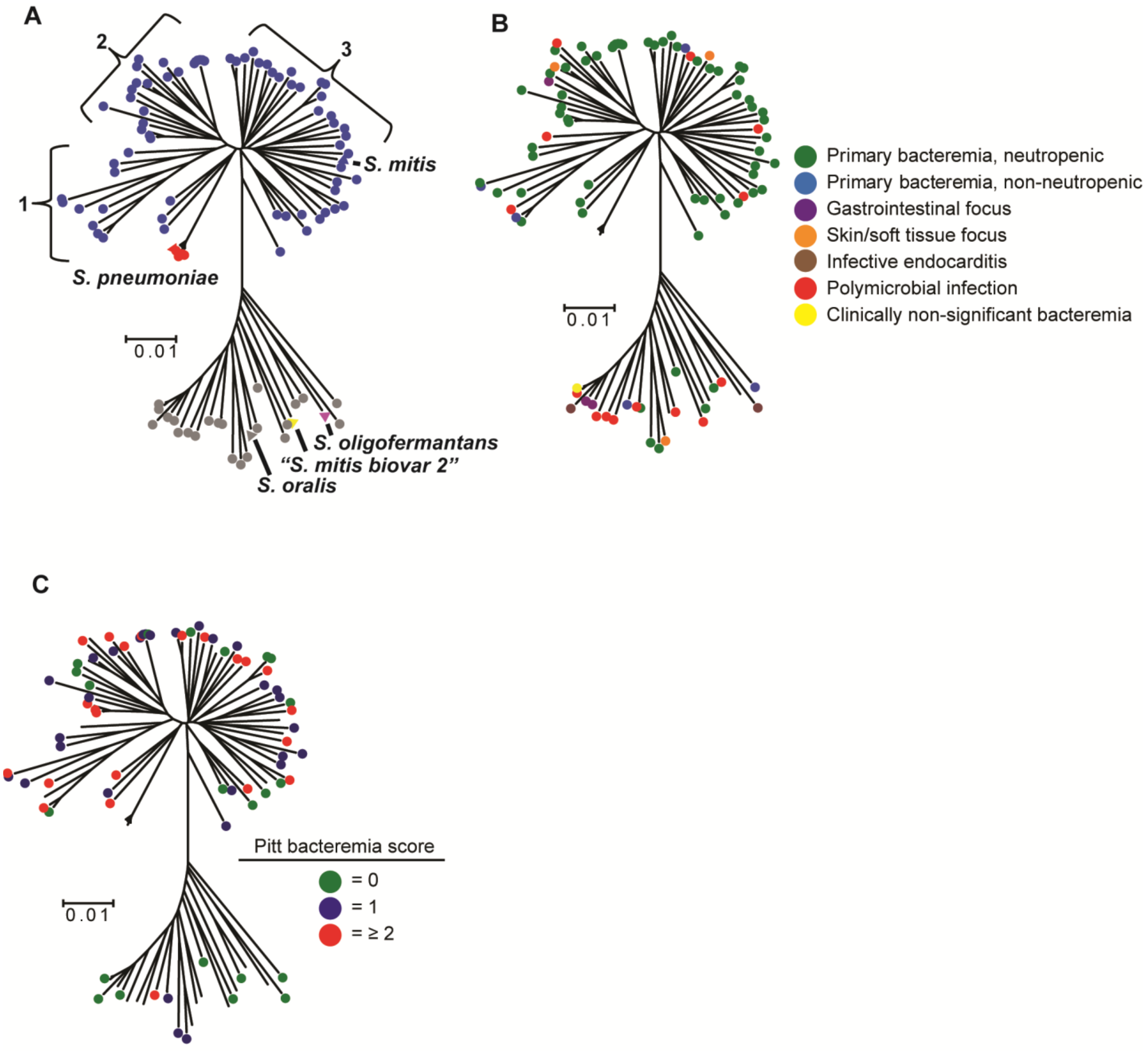

Technical Appendix 2 Figure. Multilocus sequence analysis (MLSA) and clinical correlates of *Streptococcus mitis* and *S. oralis* strains. All panels show neighbor-joining trees of *S. mitis* and *S. oralis* strains derived from MLSA analysis; the scale bar indicates the genetic distance. A) Type viridans group streptococci strains (SK142 for *S. mitis*; SK23 for *S. oralis*; and SK1136 for *S. oligofermantans*), 5 *S. pneumoniae* strains, and strain VS56 (previously characterized as an *S. mitis* biovar 2 strain) are labeled for reference purposes. Numbers 1–3 indicate *S. mitis* clusters. B) Clinical presentation for strains in the study cohort as described in the figure legend. C) Pitt bacteremia scores for strains in the study cohort as described in the figure legend. Scores could not be assigned for patients with polymicrobial infection, and thus no circle is present for strains from these patients.
